# Altered Cofactor Preference of Thermostable StDAPDH by a Single Mutation at K159

**DOI:** 10.3390/ijms21051788

**Published:** 2020-03-05

**Authors:** Xiuzhen Gao, Qinyuan Ma, Huihui Song, Xinming Sun, Zhiyun Li, Mingfei Liu

**Affiliations:** 1 School of Life Science, Shandong University of Technology, Zibo 255000, China; 17120701077@stumail.sdut.edu.cn (H.S.); 17120701080@stumail.sdut.edu.cn (X.S.); 17120701079@stumail.sdut.edu.cn (Z.L.); 17120701029@stumail.sdut.edu.cn (M.L.); 2 Key Laboratory of Industrial Fermentation, Ministry of Education; Tianjin Key Lab of Industrial Microbiology, College of Biotechnology, Tianjin University of Science and Technology, Tianjin 300457, China; ma_qinyuan@mail.tust.edu.cn

**Keywords:** *meso*-diaminopimelate dehydrogenase, D-amino acid, thermostable enzyme, cofactor engineering, molecular dynamics simulations

## Abstract

D-amino acid production from 2-keto acid by reductive amination is an attractive pathway because of its high yield and environmental safety. StDAPDH, a *meso*-diaminopimelate dehydrogenase (*meso*-DAPDH) from *Symbiobacterium thermophilum*, was the first *meso*-DAPDH to show amination of 2-keto acids. Furthermore, StDAPDH shows excellent thermostability compared to other *meso*-DAPDHs. However, the cofactor of StDAPDH is NADP(H), which is less common than NAD(H) in industrial applications. Therefore, cofactor engineering for StDAPDH is needed. In this study, the highly conserved cofactor binding sites around the adenosine moiety of NADPH were targeted to determine cofactor specificity. Lysine residues within a loop were found to be critical for the cofactor specificity of StDAPDH. Replacement of lysine with arginine resulted in the activity of pyruvic acid with NADH as the cofactor. The affinity of K159R to pyruvic acid was equal with NADH or NADPH as the cofactor, regardless of the mutation. Molecular dynamics simulations revealed that the large steric hindrance of arginine and the interaction of the salt bridge between NADH and arginine may have restricted the free movement of NADH, which prompted the formation of a stable active conformation of mutant K159R. These results provide further understanding of the catalytic mechanism of StDAPDH and guidance for the cofactor engineering of StDAPDH.

## 1. Introduction

The *meso*-diaminopimelate dehydrogenase (*meso-*DAPDH) from *Symbiobacterium thermophilum* IAM14863 (StDAPDH) was the first *meso*-DAPDH reported to catalyze the asymmetric amination of 2-keto acids, with consequent D-amino acid production [1]. D-amino acids are used in the pharmaceutical industry, foods, and cosmetics [2,3]. Among the different D-amino acid bioproduction methods, asymmetric amination with 2-keto acids as starting materials has several advantages, such as a one-step reaction, a theoretical yield of 100%, and environmental safety [2]. For instance, Akita et al. established an efficient system using the *meso*-DAPDH from *Ureibacillus thermophaericus* strain A1 (UtDAPDH) to produce D-branched-chain amino acids with a yield and optical purity > 99% [4]. 

The utilization of thermostable enzymes can decrease the cost of process control and the risk of contamination, and increase the solubility of the substrate and the reaction rates [5,6]. Therefore, thermostable enzymes are of interest for industrial applications. Recently, Akita et al. focused on the screening of more stable *meso*-DAPDHs for industrial production of D-amino acids. Finally, UtDAPDH was found to be stable after incubation for 30 min at 60 °C [7]. Divergent evolution has been found in the *meso*-DAPDH family [8]. All members of the type II group, which is represented by StDAPDH, show obvious catalytic activity with 2-keto acids. UtDAPDH belongs to type I. Five substitutions were introduced into UtDAPDH in order to create its ability to catalyze 2-keto acids [9,10]. As reported, StDAPDH is currently the most thermostable member of both groups. *S. thermophilum* is a syntrophic bacterium and its growth is dependent on co-culture with *Bacillus* [11,12,13]. The optimal culture temperature of strain T is 60 °C [11], which may explain why StDAPDH from the original strain can maintain 94% activity when incubated at 70 °C for 1 h [1]. The combination of its substrate spectrum and thermostability indicate that StDAPDH has great potential in future enzyme engineering and industrial applications [1].

**Figure 1:**
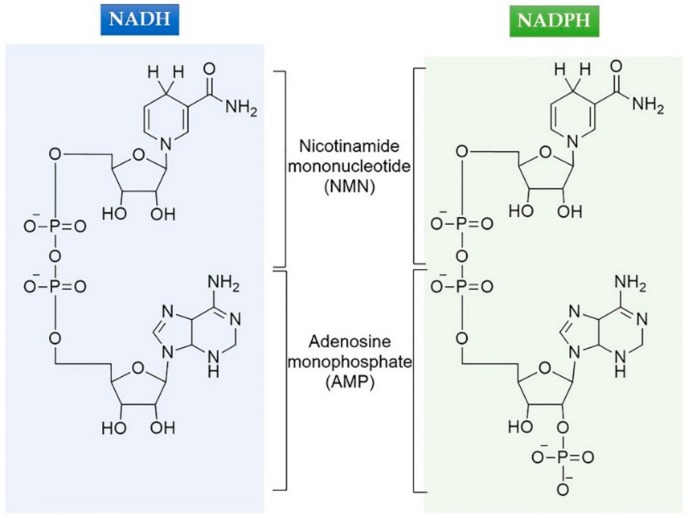
Chemical structures of NADH and NADPH.

In nature, *meso*-DAPDHs catalyze the reversible oxidative deamination of *meso*-2,6-diaminopimelate (*meso*-DAP), which is an NADP^+^-dependent process [1,14,15,16]. The sole structural difference between NAD(H) and NADP(H) is an additional phosphate group at the 2‘-hydroxyl group of the adenosine monophosphate (AMP) moiety of NADP(H) (Figure 1). However, compared to NADP^+^/NADPH, NAD^+^/NADH has some advantages in industrial applications. Firstly, NADH is less expensive than NADPH [17,18,19]. The cost of NADH is USD 126/g, while the cost of NADPH is USD 1330/g (Sigma-Aldrich, 2020 catalog). Therefore, various oxidoreductase reactions have been performed to develop regeneration systems for NAD(P)(H) [20,21,22]. For example, glucose dehydrogenase has been coupled for the synthesis of D-amino acids by *meso*-DAPDHs [4,23]. Secondly, NADH is more stable than NADPH [19,24,25]. Therefore, cofactor engineering, for enzymes such as alcohol dehydrogenase and xylose reductase, has been of great interest to various research groups [5,26,27,28]. The seminal work on cofactor engineering was completed by Scrutton et al. in 1990, in which, Arg198 and Arg204 were determined to be crucial to the cofactor specificity of glutathione reductase and mutations with a marked preference for NAD^+^ were obtained [29]. However, there is little research reported on the cofactor engineering of *meso*-DAPDHs.

In the present study, residue K159 was found to be the key residue for the cofactor specificity of StDAPDH. Molecular dynamics (MD) simulations were performed to investigate the underlying molecular mechanisms. 

## 2. Results and Discussion

### 2.1. Selection of Mutations

Divergent evolution has been reported in the *meso*-DAPDH family [8]. Amino acid residues V14, V68, P69, T70, S90, V156, and K159 of StDAPDH have been reported in our previous study [30]. These residues were predicted as NADP^+^-binding residues and are highly conserved during the evolution of type II *meso*-DAPDHs, but they differ from the residues in type I *meso*-DAPDHs. In the representative member of the type I group, *meso*-DAPDH from *Corynebacterium glutamicum* ATCC13032, the allelic NADP^+^-binding residues are L14, M66, G67, S68, T88, D154, and R157. Therefore, V14L, V68M, P69G, T70S, S90T, V156D, and K159R mutant proteins were constructed to investigate the roles of these residues during catalysis by StDAPDH. Since these seven residues are highly conserved NADP^+^-binding sites, we wanted to determine whether they were responsible for cofactor specificity.

**Figure 2:**
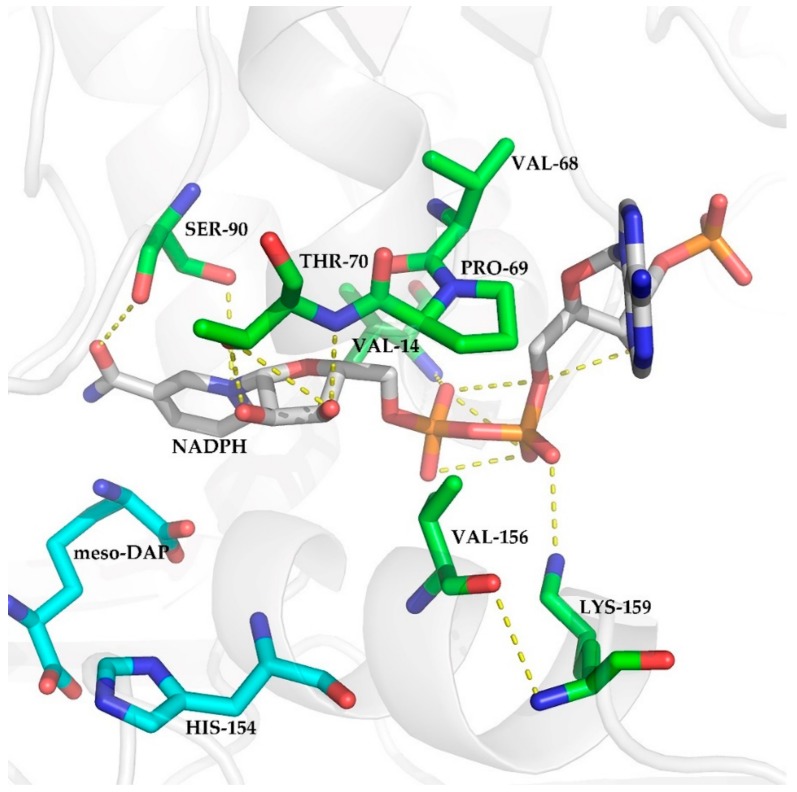
Polar contacts between NADP^+^ and residues V14, V68, P69, T70, S90, V156, and K159.

It has been reported that during catalysis by StDAPDH, H154 assists a water molecule to attract the amino acid intermediate, which is formed by hydride transfer from the Cα of *meso*-DAP to the C4N of the nicotinamide ring of NADP^+^ [30]. This indicates that the orientation of the nicotinamide ring of NADP^+^ plays a key role in catalysis by *meso*-DAPDH. Although the adenosine ring is distal from the enzyme‘s catalytic center, it is thought to have an enormous influence on enzyme activity, including kinetics and substrate specificity [31,32,33]. There have been several cofactor engineering studies focused on the residues around the AMP moiety [5,34,35]. Figure 2 shows the polar contacts between NADP^+^ and the seven residues in StDAPDH (PDB ID: 3wbf [36]). Among these polar contacts, there was only one H-bond interaction between V156 and K159. V14 and K159, which showed polar contacts with the AMP moiety, were selected for subsequent experiments.

### 2.2. Determination of Kinetic Parameters with NADH as Cofactor.

After the preparation of wild-type and mutant proteins, NADH was chosen as the cofactor to determine the kinetic parameters for pyruvic acid. As shown in Table 1, when NADH was used, the wild-type enzyme and the V14L mutant showed no catalytic activity with regard to pyruvic acid, which indicated that they were essentially specific for NADPH. By contrast, K159R was not entirely NADPH specific. The apparent K_M_ value of K159R with pyruvic acid, with NADH as the cofactor, was approximately the same as the K_M_ values of the wild-type enzyme and K159R, with NADPH as the cofactor. Therefore, this mutation resulted in an increased affinity for pyruvic acid, with NADH as the cofactor. The k_cat_/K_M_ ratio of K159R with pyruvic acid was higher than that of wild-type with NADH as the cofactor. These results suggested that K159 was a major determinant of the cofactor specificity of StDAPDH. However, the overall efficiency of K159R was significantly lower than that of the wild-type enzyme with NADPH as the cofactor, mainly due to the low turnover number (*k*_cat_). 

### 2.3. MD Simulation Analysis

To elucidate the molecular determinants of K159R on cofactor specificity, MD simulations of the protein-NADH complexes, including the native and K159R mutant proteins were performed for 100 ns. 

#### 2.3.1. Root Mean Square Deviation Analysis

**Figure 3:**
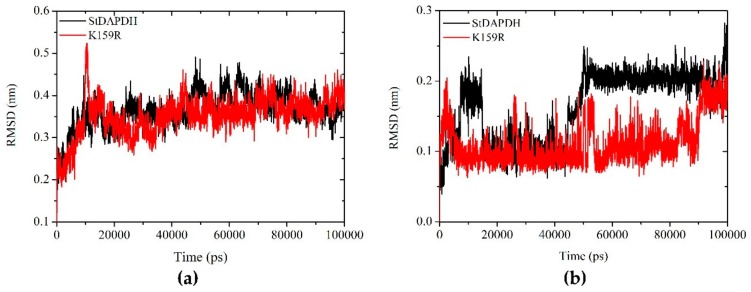
Root mean square deviation (RMSD) analysis of (**a**) proteins and (**b**) NADH of wild-type (black) and K159R (red) enzymes over a 100 ns simulation period.

Root mean square deviation (RMSD) analyses of the proteins and NADH are shown in Figure 3. The backbone of both wild-type and K159R proteins achieved a stable conformation at 20 ns and remained stable until 100 ns (Figure 3a). This indicates that the binding of NADH did not cause major changes in the structure of the enzyme. These trajectories were utilized for the further analysis.

For NADH (Figure 3b), whether combined with the wild-type or mutant enzyme, the Cα atom of NADH achieved stability at 20 ns and remained stable until 40 ns. After that, NADH bound to the wild-type enzyme showed a higher RMSD deviation compared to NADH bound to K159R, especially between 50 and 90 ns. NADH binding to K159R was stable from 20 ns, but at 90 ns, it showed some deviation. These results suggested that, compared to K159R, the wild-type enzyme was less able to form stable conformations with NADH.

#### 2.3.2. Root Mean Square Fluctuation Analysis

**Figure 4:**
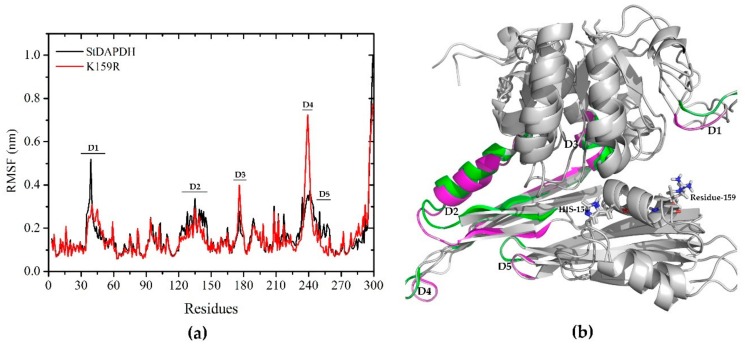
Root mean square fluctuation (RMSF) analysis (**a**) of wild-type (black) and K159R mutant (red) proteins. Residues and corresponding secondary structures (**b**) showing differences in fluctuation are marked as D1, D2, D3, D4, and D5. Green: wild-type; magenta: K159R.

In order to evaluate the plasticity of each residue of the wild-type and K159R enzymes, the root mean square fluctuation (RMSF) of the protein Cα atom was analyzed with respect to the starting structures. As shown in Figure 4a, the overall fluctuation of wild-type and mutant enzymes showed no significant differences. Residue 159 was significantly rigid in both systems. However, the K159R mutant showed higher rigidity in residues 35-39 (marked as “D1” in Figure 4), 123-146 (D2), and 243-259 (D3) and higher fluctuation in residues 175-178 (D4) and 237-241 (D5). According to the alignment of *meso*-DAPDH structures, residues 35-39 formed a loop between α2 and β2 and was located in the dinucleotide-binding domain. Residues 237-241, 123-146, and 243-259 formed loops between β12 and β13, the β-sheet β7, and β13, respectively, and were located in the polymerization domain. Residues 175-178 formed helix α10, located in the C-terminal domain [37]. For *meso*-DAPDHs, the C-terminal domain was responsible for substrate binding, while the N-terminal domain was involved in nucleotide binding [38]. As shown in Figure 4b, amino acid residues around the substrate-binding pocket showed obvious changes in flexibility, which indicated that the mutation of position 159 may reshape the active conformation of the enzyme for cofactor binding.

#### 2.3.3. Conformational Analysis

To determine whether the global change in structure improved the binding conformation of NADH, the conformations of NADH bound to wild-type and K159R mutant enzymes were compared.

**Figure 5:**
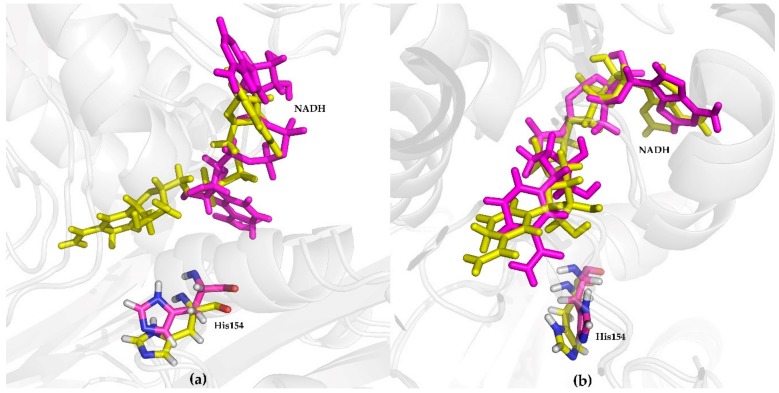
Conformational analysis of (**a**) wild-type and (**b**) K159R mutant proteins. Structure at 0 ns (yellow); structure at 100 ns (magenta).

As stated above, during MD simulations, NADH bound to the wild-type protein was less stable than NADH bound to the K159R mutant protein. Therefore, we first analyzed the orientation of NADH at 0 ns and 100 ns (Figure 5). At 0 ns, NADH showed almost the same conformation and was oriented in the substrate binding pocket in both the wild-type and mutant proteins. After 100 ns, the nicotinamide ring of NADH in the K159R mutant remained in a similar orientation (Figure 5b), but NADH in the wild-type protein was away from the catalytic site and subsequently, showed a conformational change (Figure 5a). During the first step of the catalytic process of *meso*-DAPDHs, the enzyme has an open conformation and binds NADP(H), without conformational changes. Subsequently, the substrate enters the binding pocket and the enzyme closes [38]. Therefore, the wild-type enzyme cannot bind NAD(H) following the binding of the substrate, but the K159R mutant can do so. This was in agreement with the results of the RMSD analysis of NADH.

**Figure 6:**
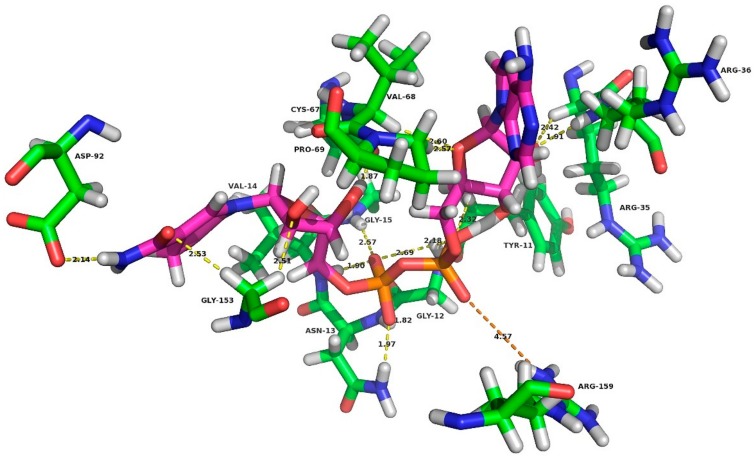
Interactions between NADH and residues within 5 Å in the K159R mutant. H-bond: yellow; salt bridge: orange.

Secondly, for the final conformation of the K159R mutant enzyme, interactions between NADH and residues within 5Å were analyzed to understand the molecular mechanisms. As shown in Figure 6, when lysine at position 159 was mutated to arginine, a salt bridge formed between R159 and atom O2A of the AMP moiety of NADH. Moreover, 15 hydrogen bond interactions were found between NADH and residues of the K159R mutant. As shown in our previous study, arginine was more easily able to form a salt bridge interaction than lysine [30]. In conclusion, the likely mechanism may be that, the larger steric hindrance of arginine compared to lysine and the formation of a salt bridge stabilized the active conformation of the enzyme when NADH was used as the cofactor.

Overall, in this study, we identified the key role of the amino acid residue at position 159 in determining the cofactor specificity of thermostable StDAPDH. The K159R mutant was found to have equal affinity to pyruvic acid with NADH or NADPH as the cofactor. However, the catalytic efficiency was still too low for industrial applications. Future research should focus on improving catalytic performance and further broadening the substrate spectrum with NADH as the cofactor. 

## 3. Materials and Methods 

### 3.1. Materials and Strains

NADH was purchased from Sigma-Aldrich (St Louis, MO, USA). Pyruvic aid and NH_4_Cl were purchased from Sinopharm Chemical Reagent Beijing Co. Ltd. (Beijing, China). The protein purification kit was purchased from Beaver Bioscience (Suzhou, China). Reagents for molecular biology were purchased from Sangon Biotech Co., Ltd. (Shanghai, China).

The K159R mutant was constructed in our previous study [30] following the protocol of whole-plasmid polymerase chain reaction in [8]. The bacterial strains producing wild-type and K159R mutant StDAPDH were maintained in our lab. 

### 3.2. Overexpression and Purification of Proteins

Both wild-type and K159R mutant proteins were overexpressed in *Escherichia coli* BL21(DE3)plys using a previously published protocol [1]. Cells were harvested by centrifugation and then disrupted by sonication (Scientz, Ningbo, China). Purified proteins were prepared by nickel chelate affinity chromatography using previously published protocols [8], which was newly cited by reference [30] and [39].

### 3.3. Determination of Kinetic Constants

Initial reaction rates were determined by monitoring the decrease in absorbance at 340 nm, which corresponds to the consumption of NADH. Kinetic parameters were determined with the concentrations of pyruvic acid varied from 0.2 mM to 20 mM using previously described protocols [39], except with NADH as the cofactor. Kinetic parameters were calculated by non-linear fitting the reaction rates versus concentrations of pyruvic acid [39].

### 3.4. MD Simulations

MD simulations were performed using the GROMACS2018.2 package [40]. The chain A coordinates of wild-type StDAPDH (PDB ID: 3wbf) were used for the construction of the protein-NADH complex *in situ*. 

The force fields Amberff99SB-ildn and gaff were applied to the protein structure and ligand, respectively [41]. The complexes were immersed in a cubic box with sufficient distance between the protein and box edges and then solvated with a TIP39 water model using the “genbox” tool [42]. Two Na^+^ were added to neutralize the system. Before equilibration, each complex was subjected to energy minimization using the steepest descent method with 10,000 steps [43] and position-restrained MD simulation for 100 ps. Equilibrations, including constant number of particles, volume, and temperature (NVT) and constant number of particles, pressure, and temperature (NPT) were then performed. NVT and NPT steps were performed for 100 ps at 298.15 K and 1 bar to stabilize the temperature and pressure of the system. Finally, a 100 ns production MD with a 2 fs step was initiated to analyze the changes in the system. Coordinates were recorded every 20 ps to construct the trajectory. Long-range electrostatic interactions were evaluated using the particle mesh Ewald method.

### 3.5. Analysis of Molecular Dynamics Trajectories

The RMSD and RMSF of the protein-NADH complex were analyzed using MD trajectories, with the built-in tools of the GROMACS package. RMSD and RMSF plots were constructed using Origin8.5 software. Visualization analyses were performed using PyMOL (http://pymol.sourceforge.net/). 

## 4. Conclusions

StDAPDH, the *meso*-DAPDH from *S. thermophilum*, is the most thermostable DAPDH currently known. It is NADP^+^-dependent and shows catalytic activity with 2-keto acids. The present study reported the altered cofactor specificity of StDAPDH from NADPH to both NADH and NADPH by the replacement of K159 with arginine and proposed an underlying mechanism. Although K159 is highly conserved among type II *meso*-DAPDHs, these results indicated that conserved amino acid residues can be chosen as targets for cofactor engineering. Taken together, the results of this study give us a deeper understanding of the catalytic mechanism of StDAPDH and provide a starting point for the engineering of *meso*-DAPDHs with NADH preference for the production of D-amino acids.

## Figures and Tables

**Table 1:** Kinetic parameters of wild-type and mutant StDAPDH with pyruvic acid as the substrate

Enzyme	K_M_ (mM)	*k*_cat_ (s^–1^)	*k*_cat_/K_M_ (mM^-1^·s^–1^)	Cofactor
StDAPDH	ND ^1^	ND	ND	NADH
	8.86 ± 0.45	7.61 ± 0.18	0.86	NADPH ^2^
V14L	ND	ND	ND	NADH
	10.08 ± 0.96	1.63 ± 0.08	0.16	NADPH ^2^
K159R	8.04 ± 0.85	1.34 ± 0.20	0.17	NADH
	7.58 ± 0.45	8.07 ± 0.21	1.06	NADPH ^2^

^1^ Not detectable. ^2^ Data from our previous study [30].

## Abbreviations

NADP(H)	Nicotinamide adenine dinucleotide phosphate
NAD(H)	Nicotinamide adenine dinucleotide
AMP	Adenosine monophosphate
NMN	Nicotinamide mononucleotide
*meso-*DAPDH	*meso*-Diaminopimelate dehydrogenase
StDAPDH	meso-DAPDH from Symbiobacterium thermophilum IAM14863
UtDAPDH	meso-DAPDH from Ureibacillus thermophaericus strain A1
*meso*-DAP	*meso*-2,6-Diaminopimelate
MD	Molecular dynamics
NVT	Constant number of particles, volume, and temperature
NPT	Constant number of particles, pressure, and temperature

## References

[1] Gao X., Chen X., Liu W., Feng J., Wu Q., Hua L., Zhu D. (2012). A novel *meso*-diaminopimelate dehydrogenase from *Symbiobacterium thermophilum*: Overexpression, characterization, and potential for D-amino acid synthesis. Appl. Environ. Microbiol..

[2] Gao X., Ma Q., Zhu H. (2015). Distribution, industrial applications, and enzymatic synthesis of D-amino acids. Appl. Microbiol. Biotechnol..

[3] Marcone G.L., Rosini E., Crespi E., Pollegioni L. (2020). D-amino acids in foods. Appl. Microbiol. Biotechnol..

[4] Akita H., Suzuki H., Doi K., Ohshima T. (2013). Efficient synthesis of D-branched-chain amino acids and their labeled compounds with stable isotopes using D-amino acid dehydrogenase. Appl. Microbiol. Biotechnol..

[5] Chen H., Zhu Z., Huang R., Zhang Y.-H.P. (2016). Coenzyme engineering of a hyperthermophilic 6-phosphogluconate dehydrogenase from NADP to NAD with its application to biobatteries. Sci. Rep..

[6] Antranikian G., Vorgias C.E., Bertoldo C. (2005). Extreme environments as a resource for microorganisms and novel biocatalysts. Adv. Biochem. Eng. Biotechnol..

[7] Akita H., Fujino Y., Doi K., Ohshima T. (2011). Highly stable *meso*-diaminopimelate dehydrogenase from an *Ureibacillus thermosphaericus* strain A1 isolated from a Japanese compost: Purification, characterization and sequencing. AMB Express.

[8] Gao X., Zhang Z., Zhang Y.n., Li Y., Zhu H., Wang S., Li C. (2017). A newly determined member of the *meso*-diaminopimelate dehydrogenase family with a broad substrate spectrum. Appl. Environ. Microbiol..

[9] Akita H., Doi K., Kawarabayasi Y., Ohshima T. (2012). Creation of a thermostable NADP^+^-dependent D-amino acid dehydrogenase from *Ureibacillus thermosphaericus* strain A1 *meso*-diaminopimelate dehydrogenase by site-directed mutagenesis. Biotechnol. Lett..

[10] Akita H., Hayashi J., Sakuraba H., Ohshima T. (2018). Artificial thermostable D-amino acid dehydrogenase: Creation and application. Front. Microbiol..

[11] Suzuki S., Horinouchi S., Beppu T. (1988). Growth of a Tryptophanase-producing thermophile, *Symbiobacterium thermophilum* gen. nov., sp. nov., is dependent on co-culture with a *Bacillus* sp.. J. Gen. Microbiol..

[12] Ueda K., Ohno M., Yamamoto K., Nara H., Mori Y., Shimada M., Hayashi M., Oida H., Terashima Y., Nagata M. (2001). Distribution and diversity of symbiotic thermophiles, *Symbiobacterium thermophilum* and related bacteria, in natural environments. Appl. Environ. Microbiol..

[13] Ohno M., Okano I., Watsuji T.-O., Kakinuma T., Ueda K., Beppu T. (1999). Establishing the independent culture of a strictly symbiotic bacterium *Symbiobacterium thermophilum* from its supporting *Bacillus* strain. Biosci. Biotechnol. Biochem..

[14] Misono H., Ogasawara M., Nagasaki S. (1986). Purification and properties of *meso*-diaminopimelate dehydrogenase from *Brevibacterium* sp.. Agric. Biol. Chem..

[15] White P. (1983). The essential role of diaminopimelate dehydrogenase in the biosynthesis of lysine by *Bacillus sphaericus*. J. Gen. Microbiol..

[16] Misono H., Togawa H., Yamamoto T., Soda K. (1976). Occurrence of *meso*-α,ε-diaminopimelate dehydrogenase in *Bacillus sphaericus*. Biochem. Biophys. Res. Commun..

[17] Woodyer R., van der Donk W.A., Zhao H. (2003). Relaxing the nicotinamide cofactor specificity of phosphite dehydrogenase by rational design. Biochemistry.

[18] Rollin J.A., Tam T.K., Zhang Y.H.P. (2013). New biotechnology paradigm: Cell-free biosystems for biomanufacturing. Green Chem..

[19] Banta S., Swanson B.A., Wu S., Jarnagin A., Anderson S. (2002). Alteration of the specificity of the cofactor-binding pocket of *Corynebacterium* 2,5-diketo-D-gluconic acid reductase A. Protein Eng..

[20] Liu W., Wang P. (2007). Cofactor regeneration for sustainable enzymatic biosynthesis. Biotechnol. Adv..

[21] Chenault H.K., Whitesides G.M. (1987). Regeneration of nicotinamide cofactors for use in organic synthesis. Appl. Biochem. Biotechnol..

[22] Liese A., Seelbach K., Wandrey C. (2006). Industrial Biotransformations.

[23] Vedha-Peters K., Gunawardana M., Rozzell J.D., Novick S.J. (2006). Creation of a broad-range and highly stereoselective D-amino acid dehydrogenase for the one-step synthesis of D-amino acids. J. Am. Chem. Soc..

[24] Wu J.T., Wu L.H., Knight J.A. (1986). Stability of NADPH: Effect of various factors on the kinetics of degradation. Clin. Chem..

[25] Wong C.-H., Whitesides G.M. (1981). Enzyme-catalyzed organic synthesis: NAD(P)H cofactor regeneration by using glucose-6-phosphate and the glucose-5-phosphate dehydrogenase from *Leuconostoc mesenteroides*. J. Am. Chem. Soc..

[26] Cahn J.K.B., Werlang C.A., Baumschlager A., Brinkmann-Chen S., Mayo S.L., Arnold F.H. (2017). A general tool for engineering the NAD/NADP cofactor preference of oxidoreductases. ACS Synth. Biol..

[27] Campbell E., Wheeldon I.R., Banta S. (2010). Broadening the cofactor specificity of a thermostable alcohol dehydrogenase using rational protein design introduces novel kinetic transient behavior. Biotechnol. Bioeng..

[28] Zeng Q.-K., Du H.-L., Wang J.-F., Wei D.-Q., Wang X.-N., Li Y.-X., Lin Y. (2009). Reversal of coenzyme specificity and improvement of catalytic efficiency of *Pichia stipitis* xylose reductase by rational site-directed mutagenesis. Biotechnol. Lett..

[29] Scrutton N.S., Berry A., Perham R.N. (1990). Redesign of the coenzyme specificity of a dehydrogenase by protein engineering. Nature.

[30] Gao X., Ma Q., Chen M., Dong M., Pu Z., Zhang X., Song Y. (2019). Insight into the highly conserved and differentiated cofactor-binding sites of *meso*-diaminopimelate dehydrogenase StDAPDH. J. Chem. Inf. Model..

[31] Mesecar A.D., Stoddard B.L., Koshland D.E. (1997). Orbital steering in the catalytic power of enzymes: Small structural changes with large catalytic consequences. Science.

[32] Cahn J.K.B., Baumschlager A., Brinkmann-Chen S., Arnold F.H. (2016). Mutations in adenine-binding pockets enhance catalytic properties of NAD(P)H-dependent enzymes. Protein Eng. Des. Sel. PEDS.

[33] Maddock D.J., Patrick W.M., Gerth M.L. (2015). Substitutions at the cofactor phosphate-binding site of a clostridial alcohol dehydrogenase lead to unexpected changes in substrate specificity. Protein Eng. Des. Sel. PEDS.

[34] González-Segura L., Riveros-Rosas H., Julián-Sánchez A., Muñoz-Clares R.A. (2015). Residues that influence coenzyme preference in the aldehyde dehydrogenases. Chem. Biol. Interact..

[35] Cho H., Oliveira M.A., Tai H.-H. (2003). Critical residues for the coenzyme specificity of NAD^+^-dependent 15-hydroxyprostaglandin dehydrogenase. Arch Biochem. Biophys..

[36] Liu W., Li Z., Huang C.-H., Guo R.-T., Zhao L., Zhang D., Chen X., Wu Q., Zhu D. (2014). Structural and mutational studies on the unusual substrate specificity of *meso*-diaminopimelate dehydrogenase from *Symbiobacterium thermophilum*. ChemBioChem.

[37] Akita H., Seto T., Ohshima T., Sakuraba H. (2015). Structural insight into the thermostable NADP^+^-dependent *meso*-diaminopimelate dehydrogenase from *Ureibacillus thermosphaericus*. Acta Crystallogr. Sect. D.

[38] Wang F., Scapin G., Blanchard J.S., Angeletti R.H. (1998). Substrate binding and conformational changes of *Clostridium glutamicum* diaminopimelate dehydrogenase revealed by hydrogen/deuterium exchange and electrospray mass spectrometry. Protein Sci..

[39] Zhang Y., Ma Q., Dong M., Zhang X., Chen Y., Gao X., Song Y. (2018). Essential role of amino acid position 71 in substrate preference by *meso*-diaminopimelate dehydrogenase from *Symbiobacterium thermophilum* IAM14863. Enzym. Microb. Technol..

[40] Abraham M.J., Murtola T., Schulz R., Páll S., Smith J.C., Hess B., Lindahl E. (2015). GROMACS: High performance molecular simulations through multi-level parallelism from laptops to supercomputers. SoftwareX.

[41] Lindorff-Larsen K., Piana S., Palmo K., Maragakis P., Klepeis J.L., Dror R.O., Shaw D.E. (2010). Improved side-chain torsion potentials for the Amber ff99SB protein force field. Proteins.

[42] Mark P., Nilsson L. (2001). Structure and dynamics of the TIP3P, SPC, and SPC/E water models at 298 K. J. Phys. Chem. A.

[43] Miyamoto S., Kollman P.A. (1992). Settle: An analytical version of the SHAKE and RATTLE algorithm for rigid water models. J. Comput. Chem..

